# Dietary Proteins, Brown Fat, and Adiposity

**DOI:** 10.3389/fphys.2018.01792

**Published:** 2018-12-12

**Authors:** Lise Madsen, Lene Secher Myrmel, Even Fjære, Jannike Øyen, Karsten Kristiansen

**Affiliations:** ^1^Institute of Marine Research, Bergen, Norway; ^2^Laboratory of Genomics and Molecular Biomedicine, Department of Biology, University of Copenhagen, Copenhagen, Denmark

**Keywords:** brown adipose tissue (BAT), diet, futile cycles, high protein diets, human, obesity, mouse, weight loss

## Abstract

High protein diets have become popular for body weight maintenance and weight loss despite controversies regarding efficacy and safety. Although both weight gain and weight loss are determined by energy consumption and expenditure, data from rodent trials consistently demonstrate that the protein:carbohydrate ratio in high fat diets strongly influences body and fat mass gain per calorie eaten. Here, we review data from rodent trials examining how high protein diets may modulate energy metabolism and the mechanisms by which energy may be dissipated. We discuss the possible role of activating brown and so-called beige/BRITE adipocytes including non-canonical UCP1-independent thermogenesis and futile cycles, where two opposing metabolic pathways are operating simultaneously. We further review data on how the gut microbiota may affect energy expenditure. Results from human and rodent trials demonstrate that human trials are less consistent than rodent trials, where casein is used almost exclusively as the protein source. The lack of consistency in results from human trials may relate to the specific design of human trials, the possible distinct impact of different protein sources, and/or the differences in the efficiency of high protein diets to attenuate obesity development in lean subjects vs. promoting weight loss in obese subjects.

## Introduction

It has for long been known that dietary protein content influences energy efficiency and thereby the energy cost for weight gain (Stock, 1999). High protein diets represent a popular alternative to energy restriction for body weight maintenance and weight loss. For instance, the Atkins diet books have sold more than 45 million copies. The Atkins diet and similar diets such as the Stone-age diet claim to be effective despite *ad libitum* consumption of high energy food items, such as fatty meat, oils, and butter, as long as the intake of carbohydrates remains lower than 50 g per day. This has in part been explained by the high satiating effect of high protein meals (Veldhorst et al., 2008; Cuenca-Sánchez et al., 2015). However, pair-feeding experiments in mice strongly suggest that increased satiety and reduced energy-intake cannot fully explain why a high protein:carbohydrate ratio in high fat diets attenuates obesity development in mice (Madsen et al., 2008, 2017; Ma et al., 2011; Qin et al., 2012). Moreover, additional effects related to increased energy expenditure of diets with high protein and low carbohydrate content have been claimed in humans (Buchholz and Schoeller, 2004; Westerterp-Plantenga, 2008; Pesta and Samuel, 2014), and based on measurements in metabolic chambers it was recently demonstrated that excess energy in the form of protein stimulated 24 h energy expenditure in men and women (Bray et al., 2015).

Taking the physical laws of energy into account, both weight gain and loss are inevitably related to consumption and use of energy. Considering the proposed positive effect of high protein diets, however, it is important to note that energy from different macronutrients may be lost to a different extent by heat generated by processing. Whereas, the thermic effects of lipids and carbohydrates are reported to be within the range of 2–3 and 6–8%, respectively, the thermic effect of proteins is reported to be 25–30% (Jequier, 2002). In addition to physical activity, energy in form of ATP may furthermore be lost in so-called “futile cycles” where two opposing metabolic pathways, such as synthesis and degradation of proteins and esterification of fatty acids and lipolysis of triacylglycerols are operating simultaneously. Energy may also be lost to the environment in form of heat via the action of uncoupling protein 1 (UCP1), present in brown and brown-like adipocytes termed BRITE (Petrovic et al., 2010) or beige adipocytes (Ishibashi and Seale, 2010; Wu et al., 2012), which uncouples oxidative phosphorylation by dissipating the proton gradient across the inner mitochondrial membrane. Historically, UCP1 was identified as the protein responsible for uncoupled respiration and heat generation in interscapular brown adipose tissue (iBAT), and cold exposure or administration of β-adrenergic agonists was subsequently reported to induce expression of UCP1 in formally white adipose tissue (WAT), especially in subcutaneous inguinal white adipose tissue (iWAT), a process termed browning, and UCP1 was considered essential for non-shivering thermogenesis and increased energy expenditure in response to cold (for a review see Cannon and Nedergaard, 2004).

Further, recent research has demonstrated additional UCP1-independent mechanisms increasing thermogenesis and energy expenditure via creatine-driven substrate cycling (Kazak et al., 2015, 2017; Bertholet et al., 2017) or Ca^2+^ cycling via the sarco/endoplasmatic reticulum Ca^2+^-ATPase 2b (SERCA2b) and the ryanodine receptor (Ikeda et al., 2017). Hence, by activation of these mechanisms it is possible to consume more energy without an accompanying weight gain. Remaining questions in this context are how such increased energy expenditure escapes the normal regulatory mechanisms aiming at maintaining energy balance, and how intake of high protein diets possibly affects this normally finely tuned homeostatic balance. So far, no comprehensive answers to these important questions have been presented, but clearly approaches to provide such answers are warranted.

Here we review mechanisms by which high protein diets may modulate energy metabolism including the possible role of activating brown and BRITE adipocytes, futile cycles, and UCP1-independent mechanisms. We also review recent data showing how the gut microbiota may impact on energy expenditure. We further discuss the lack of consistency in human trials in relation to the rodent trials demonstrating a huge difference in the potential of different protein sources to attenuate obesity development.

## Rodent Trials

### High Protein Diets, Attenuation of Obesity, and Weight Loss in Rodents

A number of rodent studies by us and others has demonstrated that a high protein:carbohydrate ratio prevents high fat diet induced obesity with an accompanied reduced feed efficiency (Marsset-Baglieri et al., 2004; Morens et al., 2005; Pichon et al., 2006; Madsen et al., 2008; Ma et al., 2011; Freudenberg et al., 2012, 2013; McAllan et al., 2014). For instance, whereas mice fed a high fat diet with low protein:carbohydrate ratio gained 14.7 g body weight per Mcal eaten, mice fed an isocaloric high fat diet with a high protein:carbohydrate ratio gained only 2.2 g body weight per Mcal eaten (Madsen et al., 2008).

**Figure 1:**
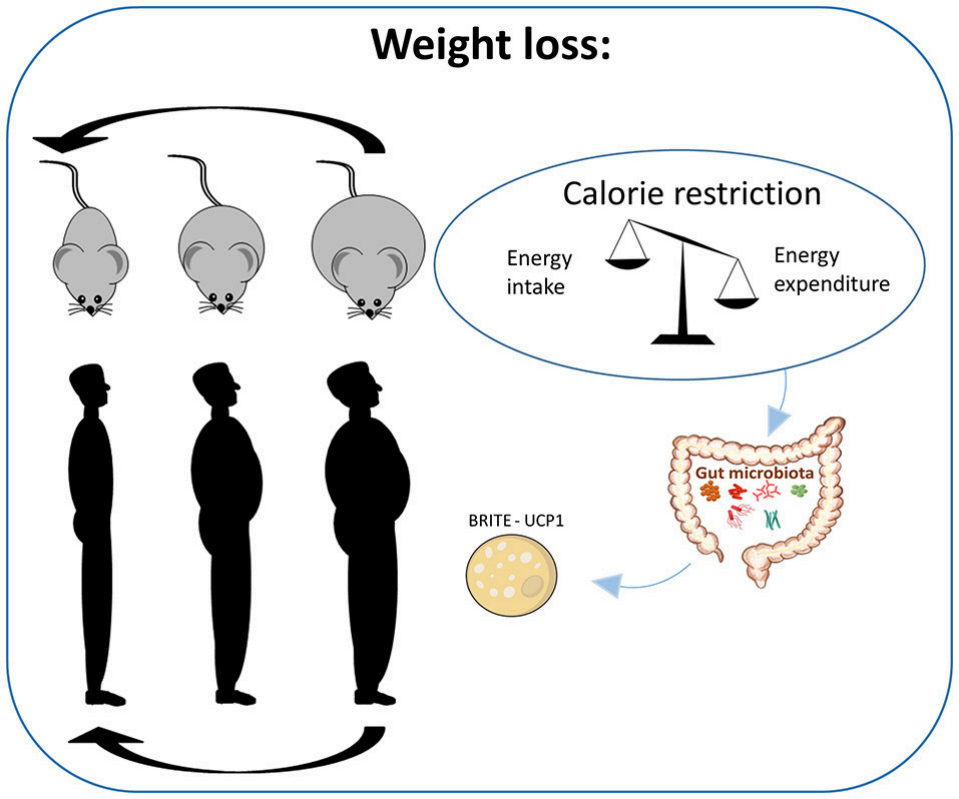
Caloric restriction and weight loss in obese individuals. Human studies involving reduced energy intake suggest calorie restriction to be the most successful dietary strategy for body weight loss. In rodents much less is known regarding the induction of weight loss in obese rodents due to the limited number of studies examining dietary interventions in already obese animals. However, a mechanistic study evaluating the impact of calorie restriction in mice has demonstrated weight loss to be dependent on browning of the white adipose tissue.

The vast majority of obesity-related rodent trials have investigated attenuation of and protection against obesity development and the efficiency of different diets to reverse obesity is far less studied (Figure 1). Inevitably, caloric restriction (Gao et al., 2015), exercise, and low fat diets promote weight loss (Jung et al., 2013), and it was suggested that weight loss is accompanied by browning of the WAT (Figure 1) (Pérez-Martí et al., 2017). Information on the efficiency of high protein diets to reverse or attenuate further obesity development is very limited. One article reported that increasing the protein content using whey, soy, red meat, or milk as the protein source to 30% at the expense of carbohydrate maintaining fat content at 40% did not reduce adipose tissue mass in already obese mice, but attenuated weight gain in a protein-dependent manner (Huang et al., 2008). Thus, when obese mice were fed whey as the protein source they gained less weight than when soy or red meat was used as protein source. The authors did not measure *Ucp1* expression or energy expenditure, and they suggested that increased adiponectin and decreased appetite may explain the stabilized adiposity in whey fed mice. Using low fat diets, we have demonstrated that the protein source may be of importance in reducing obesity, as intake of a low fat diet with casein, but not pork, reduced diet-induced obesity (Liisberg et al., 2016b). Hence, although diets with a high content of casein efficiently attenuate high fat diet induced obesity, no evidence of an obesity reversing effect of high protein diets has been published.

### Anti-obesogenic Properties of Different Protein Sources in Rodents

**Figure 2:**
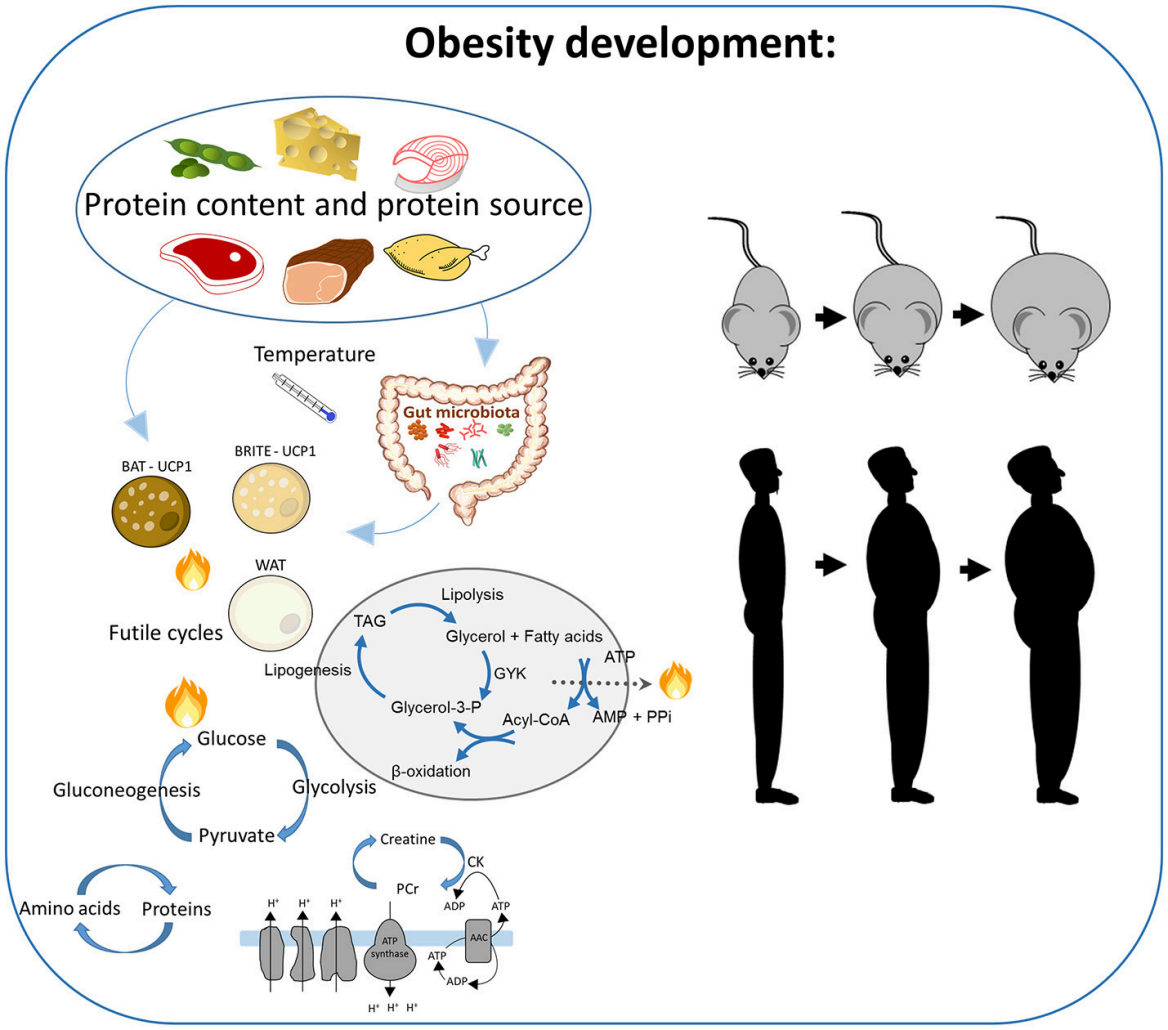
The impact of high protein intake and the source of proteins on obesity development. In rodent studies, several mechanisms are suggested to be involved, including brown adipose tissue, browning of the white adipose tissue, the gut microbiota, and different UCP1-independent futile cycles in the white adipocytes, such as simultaneous gluconeogenesis and glycolysis, protein degradation and synthesis, the futile creatine-driven substrate cycles, or the triacylglycerol breakdown and re-synthesis. The impact on obesity in response to processes dependent on UCP1 has been demonstrated to be temperature-dependent.

There is little knowledge as to how the protein source may modulate the response to high protein intake. In experiments using obesogenic diets with regular protein amounts, it has been demonstrated that protein from vegetable sources, milk protein, and proteins from seafood are less obesogenic than proteins from terrestrial animals, and this is associated with reduced energy efficiency (Figure 2) (Tastesen et al., 2014a,b; Holm et al., 2016; Liisberg et al., 2016a). However, rodent experiments, where the protein:carbohydrate ratio is increased, are in general performed using casein or whey as protein sources, and recent experiments indicate that casein and whey may not be representative. We have demonstrated that feeding obesity-prone C57BL/6J mice a high fat high protein diet using casein, soy, or filets of cod, beef, chicken or pork as protein sources led to striking differences in obesity development at thermoneutral conditions (Liisberg et al., 2016b). Casein was the most efficient protein source preventing weight gain and accretion of adipose mass, whereas mice fed high protein diets based on “white meat” (lean pork or chicken filets) gained the largest quantities of adipose tissue. Of note, iBAT in pork fed mice was composed of large unilocular “white-like” adipocytes. In casein fed mice, the classic brown adipose tissue phenotype/appearance of the iBAT was preserved with multilocular adipocytes and high UCP1 expression even at thermoneutrality. Further, the casein-induced reduction in adiposity is associated with a reversal of the obesity-induced whitening of adipocytes in iBAT and induction of UCP1 expression. Similarly, intake of high protein diets based on beef, casein and soy protein was also shown to elicit markedly different responses in relation to lipid metabolism and composition of the gut microbiota (Ijaz et al., 2018). Compared to other protein sources, casein seems to stand out either by maintaining a β-adrenergic tone or by eliciting effects via β-adrenergic independent pathways still to be identified accompanied with high expression of genes involved in a futile recycling of fatty acids, including GYK (Liisberg et al., 2016b). The GYK-dependent futile cycle may be activated without a concomitant upregulation of *Ucp1* expression and may play a key role in regulation of the metabolic flexibility, not only in adipocytes, but in the entire organism. Furthermore, it is not known if different protein sources differentially activate creatine and/or SERCA2b-dependent Ca^2+^ cycling.

### Potential Mechanisms by Which High Protein Diets May Attenuate Obesity in Rodents

High protein diets may modulate energy efficiency and thermogenesis by several mechanisms. UCP1-dependent generation of heat in BAT through non-shivering thermogenesis unquestionable plays an important role in enabling rodents to defend their body temperature (Cannon and Nedergaard, 2004) and thereby also affecting energy efficiency. Thus, several different genetically modified animal models with increased capacity for non-shivering thermogenesis are protected against diet-induced obesity (Harms and Seale, 2013). A link between UCP1 and dietary composition of macronutrients has been demonstrated in a number of studies, where rodents fed high protein diets exhibited attenuated obesity development accompanied by increased UCP1 expression and energy expenditure. Using casein-based diets with five different protein:carbohydrate ratios, it was demonstrated in mice that the body surface temperature and expression of UCP1 as well as PGC1α and DIO2, both key regulators of thermogenesis, increased in iBAT with increasing protein content in the feed. Mice fed the highest protein content gained less weight, whereas mice fed a so-called “balanced” protein to carbohydrate ratio had the highest weight gain and most pronounced adiposity (Huang et al., 2013).

In a short term experiment with rats given high protein diets, Petzke et al., demonstrated lower weight gain accompanied with higher overnight energy expenditure and oxygen consumption (Petzke et al., 2007). The expression of UCP1 was not significantly elevated in the high protein fed rats after 4 days of feeding compared with rats fed feed with lower protein content. Still, *Ucp1* mRNA levels correlated with night-time oxygen consumption, energy expenditure, and nitrogen intake. In long term experiments, the same group demonstrated increased *Ucp1* mRNA expression in iBAT by high protein feeding (Petzke et al., 2005). Also in these rats, *Ucp1* expression levels positively correlated with energy expenditure and oxygen consumption in the dark period. However, even though intake of a high dietary protein:carbohydrate ratio also attenuates obesity development during an entire life-span in mice, no increase of UCP1 expression in iBAT or iWAT was reported in these experiments (Keipert et al., 2011; Kiilerich et al., 2016),

A considerable number of experiments has failed to detect an increased level of *Ucp1* mRNA expression in iBAT despite attenuated obesity and reduced energy efficiency (Madsen et al., 2008; Ma et al., 2011; Freudenberg et al., 2012; Hao et al., 2012; Liisberg et al., 2016b). However, expression levels of *Ucp1* do not necessarily follow the thermogenic capacity (Nedergaard and Cannon, 2013), and we have in some experiments, despite no detectable induction of *Ucp1* mRNA expression, observed increased levels of UCP1 protein using immunohistochemistry and western-blotting in high protein fed mice (Liisberg et al., 2016b).

In male C57BL/6 mice, Klaus reported that development of high fat diet-induced obesity was delayed, but not prevented by increasing the protein:carbohydrate ratio (Klaus, 2005). In this experiment, a lower respiratory quotient indicated higher oxidation of fat in protein fed mice, but no differences in energy expenditure were detected. It should be noted that the findings of many studies reporting that high protein diets do not result in a significant increase in UCP1 expression or activity in iBAT are consistent with early demonstrations that unlike the activation of the central nervous system (CNS) observed with dietary carbohydrate and fat, the CNS response to dietary protein in iBAT is absent or minimal (Kaufman et al., 1986). Whether this is due to the higher protein *per se* or to lower contents of carbohydrate or fat in these diets is unknown. However, reducing the carbohydrate content, without a concomitantly increased protein content did not appear to be sufficient for increased thermogenic capacity in rats (Betz et al., 2012).

A number of studies has reported on increased expression of *Ucp1* and other markers of a brown-like phenotype in iWAT in response to increased intake of dietary protein (Madsen et al., 2008; Ma et al., 2011; Hao et al., 2012). This browning of WAT together with induction of UCP1 was associated with a reduced propensity to develop obesity accompanied with improved metabolic health (Harms and Seale, 2013). However, it has been argued that at least after cold exposure all mitochondria of iWAT only correspond to approximately 30% of the total number of mitochondria in iBAT (Shabalina et al., 2013). Still, high emergence of BRITE cells is a feature of mouse strains resistant to diet-induced obesity (Collins et al., 1997; Guerra et al., 1998). Further, the lean phenotype associated with aP2-driven expression of UCP1 is linked to increased energy dissipation in WAT (Kopecký et al., 1996b). Endogenous UCP1 expression as well as the respiration rate is actually reduced in iBAT from these mice (Kopecký et al., 1996a).

Resistance against high fat diet-induced obesity is demonstrated in several genetically modified mice associated with an increased occurrence of BRITE adipocytes in former WAT, such as mice deficient in RIP140 (Leonardsson et al., 2004), Caveolin (Razani et al., 2002), FSP27 (Toh et al., 2008), HSL (Ström et al., 2008), RB (Hansen et al., 2004; Dali-Youcef et al., 2007; Mercader et al., 2009), and p53 (Hallenborg et al., 2016). Interestingly, the thermogenic activity in iBAT of these modified strains was unchanged or reduced. It may also be noted that depletion of BRITE adipocytes by PRDM16 ablation in mice leads to moderate obesity (Cohen et al., 2014). For an extensive list of genes affecting formation of BRITE adipocytes, see (Harms and Seale, 2013). However, it is a distinct possibility that the blunted development of obesity in these studies with no detectable induction of UCP1 expression in BRITE or brown adipocytes may be caused by UCP1-independent mechanisms including creatine-driven cycles and/or SERCA2b-dependent Ca^2+^ cycling, and future studies on the possible involvement of such mechanisms in response to intake of high protein diets would clearly be of interest.

Assessing the obesogenic capacity of different diets at different housing temperatures may also contribute to an understanding of the mechanisms by which diets influence obesity development. The importance of UCP1 and diet-induced thermogenesis during high protein feeding may only be clearly manifested in mice kept at thermoneutrality (28–30°C). Cold-sensitive mice lacking UCP1 are not more obesity prone than wild type mice when fed a high fat diet and kept at 21°C (Enerback et al., 1997). However, at 30°C, the *Ucp1*-KO mice are more susceptible to obesity than their wild type littermates and become by far more obese, demonstrating the importance of UCP1 for diet-induced thermogenesis and prevention of obesity development (Feldmann et al., 2009). Compared with thermoneutrality, housing at standard vivarium temperatures, 20–22°C, leads to higher food intake required to meet the increased energy demand for thermogenesis (Cannon and Nedergaard, 2004; Fischer et al., 2018). Increasing the protein:carbohydrate ratio in high fat diets attenuate obesity development and increase UCP1 expression in mice kept at both 22°C (Madsen et al., 2008) and at thermoneutrality (Ma et al., 2011), but obesity development and feed-efficiency in mice kept at the two different temperatures have not yet been directly compared. Such comparison may be useful in determining the mechanisms by which high protein diets mediate their anti-obesogenic effects.

Whereas UCP1-dependent uncoupled respiration in iBAT is crucial during cold exposure, recent results has convincingly demonstrated that UCP1-independent thermogenesis and increased energy expenditure are associated with BRITE adipocytes (for recent reviews see Ikeda et al., 2018; Sponton and Kajimura, 2018). Thus, it is possible that such UCP1-independent processes may also contribute to the decreased energy efficiency in response to intake of high-protein diets. This notion also extends to loss of fat mass in response to caloric restriction, which has been shown to be dependent on browning (Fabbiano et al., 2016). Still, although it is evident that feeding mice high protein diets reduces energy efficiency, further investigations are required to determine the importance of energy dissipation in form of heat by activation of UCP1 or UCP1-independet processes in BRITE adipocyte, but evidence from studies on the SERCA2b-dependent Ca^2+^ cycling would suggest that the contribution of this process may account for much of the diet-induced thermogenesis in BRITE adipocytes (Ikeda et al., 2018; Sponton and Kajimura, 2018).

Temperature may also affect the process of browning by changing the composition and function of the gut microbiota. It was initially observed that germ-free mice were resistant to high fat diet-induced obesity (Bäckhed et al., 2004). Interestingly, it has been shown that antibiotics-mediated depletion of the gut microbiota promoted browning of iWAT and perigonadal WAT (pWAT) in lean and obese mice, and increased browning was also observed in germ-free mice by the Trajkovski group (Suarez-Zamorano et al., 2015). Surprisingly, the same group also reported that exposure to cold resulted in major changes in the composition and function of the gut microbiota in mice and that transplantation of the gut microbiota from cold-adapted mice to germ-free mice by co-housing increased browning of iWAT and pWAT (Chevalier et al., 2015). Similarly, the Bäckhed group reported that cold exposure drastically altered the composition of the gut microbiota accompanied by an attenuated propensity for diet-induced obesity, and the obesity-resistant phenotype could be transferred to germ-free mice by fecal transplantation (Zietak et al., 2016). However, in this case the most pronounced effect in terms of inducing UCP1 expression was observed in iBAT, and in addition, increased fatty acid oxidation was observed in the liver (Zietak et al., 2016).

Although commonly used in commercial available rodent feed, casein and whey protein, appear to possess anti-obesogenic properties (Lillefosse et al., 2014; Tastesen et al., 2014b; Pezeshki et al., 2015; Liisberg et al., 2016b; Singh et al., 2016). This may be related to the high content of the branched-chain amino acids (BCAA). BCAA is reported to increase the abundance of *Akkermansia* and *Bifidobacterium* in the gut (Yang et al., 2016). High levels of both *A. muciniphilia* (Everard et al., 2013; Shin et al., 2014) and some strains of *Bifidobacteria* (An et al., 2011; Wang et al., 2015; Li et al., 2016) have been reported to protect against diet-induced obesity.

BCAAs may also directly affect metabolism, as inclusion of BCAAs attenuates high fat diet induced obesity in rats (Newgard et al., 2009) and mice with a disrupted mitochondrial branched chain amino transferase gene exhibit chronic elevated levels of BCAAs in blood and have increased energy expenditure (She et al., 2007). The importance of BCAA is further supported by studies from Freudenberg et al. demonstrating that adding leucine to a high fat diet with regular protein content to a level matching a diet with high content of whey attenuated obesity development (Freudenberg et al., 2012, 2013). However, equimolar supplementation with alanine decreased body fat mass gain to the same extent (Freudenberg et al., 2013; Petzke et al., 2014). Hence, at least some of the observed effects are not specifically related to BCAA, but rather increased amino nitrogen consumption. Together, animal studies indicate that casein and whey have anti-obesogenic properties compared with other protein sources. Hence, studies investigating the effect of high protein diets using casein or whey may not be representative. In fact, high fat high protein diets with meat appear to promote obesity development (Pezeshki et al., 2015; Liisberg et al., 2016b; Madsen et al., 2017; Ijaz et al., 2018).

In mice fed either standard low fat diets, or high fat diets with high or low protein:sucrose ratio, the dietary fat content is a stronger driver of the composition of the gut microbiota than the protein:sucrose ratio (Kiilerich et al., 2016). However, still certain phylotypes within the *Clostridiaceae* family (*Anaerovorax, Bryantella, C. herbivorans, C. sphenoides, C. leptum*, and *C. symbiosum*) were found to characterize the gut in high protein fed mice (Kiilerich et al., 2016). Whether the accompanied high protein induced changes in energy expenditure and/or UCP1 expression are linked to the observed changes in gut microbiota composition requires further investigations. It should be mentioned, however, that a high dietary protein content in diets with regular fat content led to decreased abundances of *Akkermansia muciniphila, Bifidobacterium, Prevotella, Ruminococcus bromii*, and *Roseburia/Eubacterium rectale*, and thereby a decreased number and activity of propionate- and butyrate-producing bacteria in rats (Mu et al., 2017). This would be counteractive in terms of obesity, as *Akkermansia muciniphilia* (Everard et al., 2013; Shin et al., 2014), and short chain fatty acids are reported to attenuate diet induced obesity in mice (Lin et al., 2012; Lu et al., 2016) and have been associated with protection against diet-induced obesity and associated metabolic disorders in part by increased energy expenditure and thermogenesis (Gao et al., 2009). Thus, as high protein feeding modulates the composition of the gut microbiota, it is possible that such changes contribute to the reduced accumulation of fat, but it also remains to be investigated to what extent creatine-driven cycling or SERCA2b-dependent processes following browning contributes to these observed physiological phenotypes.

**Figure 3:**
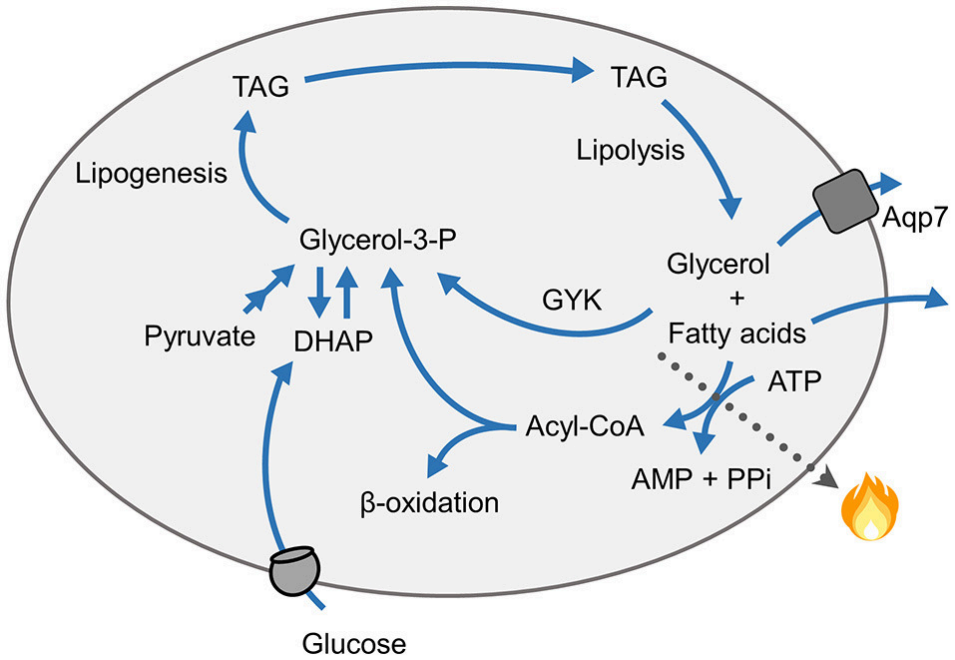
Schematics of futile cycling of triacylglycerol (TAG) breakdown and re-synthesis. Re-esterification of fatty acids into triacylglycerol requires 6 ATPs for re-synthesis of one triacylglycerol molecule following lipolysis. The re-synthesis of triacylglycerols from fatty acids also requires a supply of glycerol-3-phosphate (Glycerol-3-P), which may be produced from dihydroxyacetone-phosphate (DHAP) or originate from glucose via the glycolytic pathway, from pyruvate, or glycerol by the action of glycerol kinase (GYK).

Apart from creatine and Ca^2+^ cycling discussed above, energy may also be lost in an UCP1-independent manner by futile cycling of fatty acids. Re-esterification of fatty acids into triacylglycerol following lipolysis occurs in both humans and rodents (Figure 3). This process of triacylglycerol/fatty acid cycling is a futile cycle as 6 ATPs are required for re-synthesis of one triacylglycerol molecule. Work from Kopecky's laboratory has demonstrated that the ATP required for both triacylglycerol/fatty acid cycling as well as the associated *de novo* synthesis of fatty acids is produced by oxidative phosphorylation in white adipocytes (Kuda et al., 2018). Their work suggested that the UCP1-independent energy dissipation linked to futile triacylglycerol/fatty acid cycling may contribute to non-shivering thermogenesis as well as the observed amelioration of obesity induced by calorie restriction combined with the intake of omega-3 fatty acids. Of note, cold exposure leads to induction of genes involved in both triacylglycerol synthesis as well as *de novo* lipogenesis in WAT (Hao et al., 2010; Kiskinis et al., 2014; Gao et al., 2015; Flachs et al., 2017).

Intake of diets with high protein:carbohydrate ratio is accompanied by increased expression of genes involved in futile recycling of glycerol and fatty acids in iBAT (Liisberg et al., 2016b). Re-esterification of fatty acids into triacylglycerols within adipocytes also requires a continuous supply of glycerol-3-phosphate (Figure 3). Possibly, to avoid re-esterification of glycerol, and thereby futile cycling of triacylglycerol in adipose tissue, glycerol kinase (GYK) expression and activity is absent or low in adipose tissue. However, PPARγ activating drugs, such as thiazolinediones, induce the expression of GYK in adipocytes (Guan et al., 2002), and more studies have demonstrated that GYK expression in adipocytes is controlled by PGC-1α and PPARα (Mazzucotelli et al., 2007). In addition to increased *Ucp1* expression in eWAT, energy loss due to increased expression of GYK and other genes involved in the futile cycling of triacylglycerol breakdown and synthesis was suggested to explain the lean phenotype of mice lacking RIP140 (Leonardsson et al., 2004). Using casein as the protein source, we have observed that a high protein:carbohydrate ratio in a high fat diet is accompanied by increased expression levels of GYK and other genes involved in a futile recycling of glycerol and fatty acids in iBAT (Liisberg et al., 2016b). We have suggested that futile cycling of triacylglycerol breakdown and re-synthesis supports the enhanced uncoupled respiration and thereby plays a part in mediating the anti-obesogenic effect of certain high protein diets. Of note, the diets used by us were not carbohydrate free, and GYK-expression may be related to a more brown phenotype and possibly higher sympathetic flow.

When the intake of carbohydrates is low, the ratio of circulating insulin:glucagon is low and glucose may be provided by hepatic gluconeogenesis via activation of hepatic PGC-1α (Herzig et al., 2001; Yoon et al., 2001; Puigserver et al., 2003). Hence, when intake of carbohydrates is sufficiently low, gluconeogenesis and glycolysis may occur simultaneously. Gluconeogenesis requires a higher number of ATP molecules than provided by glycolysis. Further, a concomitant conversion of protein to metabolites that may enter gluconeogenesis may occur. Protein is converted to glucose at a cost of 4–5 kcal/g protein (Fine and Feinman, 2004), and this may contribute to reduced feed efficiency. We have earlier demonstrated that a reduced circulating insulin:glucagon ratio was accompanied by increased expression of PGC-1α and gluconeogenesis in the liver of high protein fed mice and suggested that this may contribute to the lean phenotype in these mice (Madsen et al., 2008). The increased glucagon:insulin ratio observed in mice fed a high proportion of casein may further lead to reduced insulin signaling in adipose tissue. This may be of particular importance, as studies in mice with fat-specific disruption of the insulin receptor gene have demonstrated that insulin signaling in adipocytes is crucial for development of obesity (Blüher et al., 2002). Furthermore, prevention of hyperinsulinemia is demonstrated to protect against diet-induced obesity in *Ins1*^+/−^*:Ins2*^−/−^ mice (Mehran et al., 2012). Concomitantly, enhanced cAMP-dependent signaling and PKA activation in adipose tissues may occur as we have observed increased phosphorylation of CREB and enhanced expression of the canonical cAMP-responsive genes, cAMP-responsive element modulator and cAMP-specific phosphodiesterase 4c, in both white and brown adipose tissue (Madsen et al., 2008). Hence, processes required to efficiently store fat may be inhibited.

Feed efficiency may also be reduced by simultaneous increased protein degradation and synthesis as observed in mice with disrupted branched-chain amino acid metabolism (She et al., 2007). In addition, both protein synthesis and proteolysis are energy demanding processes (Reeds et al., 1985). In men, it has been reported that repeated intake of a high protein meals after fasting led to a higher thermic response and nitrogen turnover compared to what was observed after repeated intake of carbohydrate rich meals (Robinson et al., 1990). Using theoretical estimates of ATP requirements, the authors suggested that increased protein synthesis accounted for more than 65% of the thermic response after high protein meals. An increased catabolism of amino acids requires ATP to dispose of nitrogen as urea at an energy cost of 1.33 kcal/g urea. In mice, increased intake of water and accompanied increased production of urea have been observed in high protein fed mice (Madsen et al., 2008). Interestingly, increased water intake has been associated with reduced obesity (Thornton, 2016). It is proposed that increased water intake, in addition to reducing food intake, can activate thermogenesis via release of atrial natriuretic peptide (Thornton, 2016).

Together, published results from rodent studies suggest that high protein diets can lead to increased energy expenditure by several mechanisms, including activation of brown and so-called BRITE adipocytes. As discussed above, recent findings would indicate that BRITE adipocytes seem to play a greater role than canonical iBAT and that creatine cycling, SERCA2b-dependent Ca^2+^ cycling, and/or futile cycling of fatty acids may play pivotal roles. In addition, energy loss via increased production of urea may also contribute. Furthermore, a number of secreted factors, batokines, from brown and BRITE adipocyte has been shown to positively or negative modulate brown/BRITE adipocyte differentiation and thermogenesis (Sponton and Kajimura, 2018), and also here is an interesting question whether intake of high protein diets and/or different types of protein will affect these processes as well.

## Human Trials

### High Protein Diets and Weight Loss in Humans

The effects of high protein diets in rodents have mostly been investigated in relation to prevention of obesity development. By contrast, human trials have to a larger extent examined the ability of high protein diets to induce weight loss in obese subjects, and knowledge on how increased intake of protein prevents weight gain in humans is limited. Systematic reviews and meta-analyses examining the efficiency of high protein diets to promote weight loss in humans are not consistent. A systematic review and meta-analyses of 74 randomized controlled trials (RCTs) from 2012, where diets low and high in protein content were compared, concluded that high protein diets led to a greater weight reduction after 3 months than low protein diet (Santesso et al., 2012). Wycherley et al. examining 24 trials concluded that increasing the protein content elicited modest weight loss (Wycherley et al., 2012b), whereas a systematic review by Lepe et al. concluded that the long-term effects of high-protein diets were neither consistent nor conclusive (Lepe et al., 2011). A selection of RCTs investigating the long-term effects of high-protein diets on body composition is presented in Table 1. In a number of these trials, no marked differences between the two dietary groups were observed, whereas a modest effect was observed in others. Of note, in these trials the interventions combined high protein diets with energy restriction.

**Table 1:** A selection of Randomized Controlled Trials investigating the long-term effects of high-protein diets on body weight.

**References**	**Population**	**Design**	**Intervention groups (protein, carbohydrate, fat)**	**Energy restricted (kcal day)**	**Duration (months)**	**Drop out**	**Body weight reduction pre-post (kg)**	***P*-between groups**
Skov et al., 1999	*N* = 65 (15 M, 50 F) 18–56 y BMI: 25–34 kg/m^2^	RCT, parallel	1) HP/LF (25, 45, 30 E%) 2) LP/LF (12, 58, 30 E%)	No	6	8%	1) −8.9 2) −5.1	< 0.001
Due et al., 2004	*N* = 50 19–55 y BMI: 26–34 kg/m^2^	RCT, parallel	1) HP/LF (25, 45, 30 E%) 2) LP/LF (12, 58, 30 E%)	No	24	66%	1) −6.4 2) −3.2	NS
Brinkworth et al., 2004b	*N* = 66 >60 y BMI: 27–40 kg/m^2^	RCT, parallel	1) HP/LF (30, 40, 30 E%) 2) LP/LF (15, 55, 30 E%)	1600 (8 weeks), energy balance (4 weeks), no restriction (follow-up)	15	42%	1) −3.7 2) −2.2	NS
Brinkworth et al., 2004a	*N* = 58 (13 M, 45 F) 20–65 y BMI: 27–43 kg/m^2^	RCT, parallel	1) HP/LF (30, 40, 30 E%) 2) LP/LF (15, 55, 30 E%)	1,555 (12 weeks), energy balance (4 weeks), no restriction (follow-up)	16	26%	1) −4.1 2) −2.9	NS
Layman et al., 2009	*N* = 130 (59 M, 71 F) 45 ± 1 y BMI: 32.6 ± 0.8 kg/m^2^	RCT, parallel	1) HP/LF (30, 40, 30 E%) 2) LP/LF (15, 55, 30 E%)	1,900 M, 1,700 F	12	55%	1) −10.4 2) −8.4	NS
Sacks et al., 2009	*N* = 811 51 ± 9 y BMI: 33 ± 4 kg/m^2^	RCT, parallel	1) AP/LF (15, 65, 20 E%) 2) HP/LF (25, 55, 20 E%) 3) AP/HF (15, 45, 40 E%) 4) HP/HF (25, 35, 40 E%)	−750	24	79%	1) −3.6 2) −4.5 3) −3.6 4) −4.5	NS
Larsen et al., 2011	*N* = 99 (48 M, 51 F) 58–62 y BMI: 27–40 kg/m^2^	RCT, parallel	1) HP/LF (30, 40, 30 E%) 2) LP/LF (15, 55, 30 E%)	1,530 (12 weeks), energy balance (follow-up)	12	20%	1) −2.23 2) −2.17	NS
Krebs et al., 2012	*N* = 419 (168 M, 251 F) 30–75 y BMI: 36.6 ± 6.5 kg/m^2^	RCT, parallel	1) HP/LF (30, 40, 30 E%) 2) LP/LF (15, 55, 30 E%)	−500	24	30%	1) −3.9 2) −6.0	NS
Wycherley et al., 2012a	*N* = 120 51 ± 9 y BMI: 33.0 ± 3.9 kg/m^2^	RCT, parallel	1) HP/LF (35, 40, 25 E%) 2) LP/LF (17, 58, 25 E%)	1,700	12	44%	1) −12 2) −10.9	NS

### Effects of Different Dietary Protein Sources on Body Composition

Epidemiological studies indicate that whereas diets with dairy and vegetarian protein sources protect against obesity, diets with a high proportion of meat, in particular red meat, are associated with higher weight gain (Fogelholm et al., 2012; Smith et al., 2015; Mozaffarian, 2016). A number of RCTs examining the influence of diets based on different protein sources on weight loss has been carried out (Table 2). In several of these, where the dietary intervention was combined with energy restriction (Melanson et al., 2003; Mahon et al., 2007), the protein source did not affect weight loss, and differences in design and population groups make it difficult to draw clear and firm conclusions. However, a recent position paper from MyNewGut concluded that intake of a high protein diet generally decreased body weight development, but the effects varied according to the type of dietary intervention and protein source, and that intake of a high protein diet was accompanied with changes in the gut microbiota (Blachier et al., 2018).

**Table 2:** A selection of Randomized Controlled Trials investigating the effects of diets based on different protein sources on body weight.

**References**	**Population**	**Design**	**Intervention groups (protein, carbohydrate, fat)**	**Energy restricted (kcal day)**	**Duration (weeks)**	**Body weight reduction pre-post**	***P*-between groups**
Yamashita et al., 1998	*N* = 36 (F) 40 ± 9 y BMI: 32.4 ± 5.2 kg/m^2^	Non-randomized, parallel	1) Plant (25, 50, 23 E%) 2) Red meat (25, 51, 22 E%)	1,500	16	1) −7.6 kg 2) −7.8 kg	NS
Mori et al., 1999	*N* = 63 (42 M, 21 F) 54 ± 2 y BMI: 31.6 ± 1.1 kg/m^2^	RCT, parallel	1) Fish 2) Weight-loss 3) Fish + weight loss 4) Control	−478 to 1,554 (groups 2–3)	16	1) 0.5 2) −5.2 kg 3) −5.9 kg 4) 0.1 kg	*P* < 0.05 for groups 2 and 3 vs. 1 and 4
Melanson et al., 2003	*N* = 61 (F) 43 ± 8 y BMI: 32.1 ± 3.4 kg/m^2^	RCT, parallel	1) Chicken (21, 57, 22 E%) 2) Beef (20, 55, 25 E%)	−500	12	1) −6.0 ± 0.5 kg 2) −5.6 ± 0.6 kg	NS
Mahon et al., 2007	*N* = 54 (F) 58 ± 2 y BMI: 29.6 ± 0.8 kg/m^2^	RCT, parallel	1) Beef (26, 48, 26 E%) 2) Chicken (26, 48, 26 E%) 3) Carbohydrate (16, 58, 26 E%) 4) Control (habitual diet)	−1000 (groups1–4)	9	1) −6.6 ± 2.7 kg 2) −7.9 ± 2.6 kg 3) −5.6 ± 1.8 kg 4) −1.2 ± 1.2 kg	*P* < 0.05 for groups 1–3 vs. 4, and 2 vs. 3
Thorsdottir et al., 2007	*N* = 324 (138 M, 186 F) 39 ± 5 y 30.1 ± 1.4 kg/m^2^	RCT, parallel	1) Control 2) Lean Fish 3) Fatty fish 4) Fish oil	−30% 2,694 M 2,022 F	8	M: 1) −5.3 ± 3.0 kg 2) −6.5 ± 2.8 kg 3) −7.0 ± 3.5 kg 4) −6.7 ± 3.2 kg	M: *p* < 0.05 for groups 2–4 vs. 1. All + F: NS
Liu et al., 2010	*N* = 180 (F) 56 ± 4 y BMI: 24.5 ± 3.7 kg/m^2^	Double blinded, RCT, parallel	1) Placebo (15 g milk protein) 2) Iso (15 g milk protein + 100 mg isoflavones) 3) Soy (15 g soy protein + 100 mg isoflavones)	≈2,140	26	1) −0.3 ± 2.6% 2) −0.3 ± 2.5% 3) −1.2 ± 3.0%	*P* < 0.05 for group 3 vs. 1 and 2
Aadland et al., 2015	*N* = 20 (7 M, 13 F) 50.6 ± 3.4 y BMI: 25.6 ± 0.7 kg/m^2^	RCT, cross-over	1) Lean seafood (20, 52, 28 E%) 2) Beef (20, 52, 28 E%)	717 M 538 F	2 × 4	1) −1.4 ± 0.2 kg 2) −1.5 ± 0.2 kg	NS

Inclusion of fatty fish, lean fish, or fish oil as part of an energy-restricted diet significantly increased weight loss in young overweight men (Thorsdottir et al., 2007). From these RCTs comparing the impact of different protein sources on body weight when combined with energy restriction, there is no clear evidence that one protein source is to be preferred relative to another (Figure 1). Hence, it may be speculated if the protein sources are of more importance in the habitual diets to prevent weight gain than in energy restricted diets used to achieve weight loss.

## Summary and Conclusion

The ability of high protein diets using casein or whey as the protein source to prevent weight gain is well-documented in mouse studies. The accompanying reduced feed-efficiency may be related to an increased glucagon:insulin ratio, increased uncoupled respiration and/or ATP loss by futile cycling. To what extent the reduced feed-efficiency and increased energy expenditure are mediated via the gut microbiome is not yet known. Further, intake of high protein diets may lead to reduced insulin signaling in adipose tissue. Of note, these effects appear to be restricted to diets where casein or whey are used as protein sources and evidence that high fat high protein diets are able to induce weight loss in rodents is lacking.

The effect of high protein diets on weight loss in humans is not conclusive. However, in line with the importance of the protein source in rodent studies, where obesity development is examined, epidemiological studies indicate that diets with dairy and vegetarian protein sources protect against, whereas diets with meat may promote obesity. However, when diets containing different protein sources are examined in combination with energy restriction, the protein source appears to be of little or no importance. Together, these data indicate that the dietary protein source is of greater importance in preventing weight gain than during weight reduction. It may be considered if dietary means for obesity prevention in lean persons may be different from dietary advices to obese subjects to achieve effective weight loss. Based on the different approaches in human and animal studies investigating dietary protein sources, we encourage to performing more studies in humans and animals focusing on weight gain, weight maintenance and weight loss with different dietary protein sources in order to determine the possible impact of protein source on obesity development and reversal. To what extent high protein intake in humans modulates energy expenditure via the gut microbiome, uncoupled respiration or other energy consuming futile cycles remains to be established.

## Author Contributions

LM researched data and wrote the first draft. LSM, EF, JØ, and KK contributed with text, discussion of the content, illustrations, revised, and/or edited the manuscript before submission.

### Conflict of Interest Statement

The authors declare that the research was conducted in the absence of any commercial or financial relationships that could be construed as a potential conflict of interest.
